# CD2 costimulation breaks the CD28 vs. 4-1BB tradeoff in CAR T cells

**DOI:** 10.1016/j.omton.2026.201149

**Published:** 2026-02-20

**Authors:** Avery D. Posey

**Affiliations:** 1 Department of Systems Pharmacology and Translational Therapeutics, Perelman School of Medicine, University of Pennsylvania, Philadelphia, PA, USA; 2 Corporal Michael J. Crescenz VA Medical Center, Philadelphia, PA, USA

## Main text

Chimeric antigen receptor (CAR) T cells represent the most clinically validated form of *ex vivo* somatic gene therapy. In this approach, patient T cells are genetically modified, most commonly via viral gene transfer, to stably express a synthetic receptor that redirects antigen recognition and converts target engagement into T cell activation.^1^^,^^2^ Therapeutic performance is therefore shaped not only by the choice of antigen but also by how the engineered receptor is configured and expressed, including its domain organization, surface expression level, and the extent to which signaling remains antigen-coupled.^2^ Together, these design features influence T cell expansion, differentiation, functional exhaustion, and long-term persistence *in vivo*. The clinical performance of CD19- and B cell maturation antigen (BCMA)-directed CAR T cell therapies demonstrates that engineered receptors can drive deep tumor clearance in favorable settings.^1^ However, the modular architecture of CARs also highlights a central limitation of molecular therapy: efficacy and durability depend on the cell-fate programs imposed by CAR intracellular signaling, particularly under sustained antigen exposure and a suppressive tumor microenvironment.^2^

In practice, approved CAR T cell products, as well as many clinically evaluated platforms, have relied primarily on CD28- and 4-1BB-based costimulation, consistent with the reproducible clinical activity of these architectures in B cell and plasma cell malignancies.^1^^,^^2^ CD28 and 4-1BB are frequently treated as canonical endodomain archetypes that bias CAR T cells toward distinct activation dynamics and differentiation trajectories: CD28-based receptors commonly drive faster early activation and effector differentiation, whereas 4-1BB-based receptors more frequently support longer persistence through distinct downstream signaling and metabolic programs.^3^ In advanced and aggressive tumors, engineered T cells encounter heterogeneous antigen density and prolonged stimulation in the setting of robust immunosuppression and metabolic constraint. Under these conditions, canonical endodomain biases can become liabilities, either by promoting dysfunction when signaling is strong and sustained or by limiting early cytolytic activity such that tumor burden is not sufficiently reduced.

In our recent study, we expanded the endodomain design space by evaluating the CD2 cytoplasmic tail as a CAR costimulatory endodomain.^4^ We then benchmarked its signaling and functional consequences against matched CD28- and 4-1BB-based architectures in anti-mesothelin and anti-Tn-MUC1 CAR platforms. CD2 is a bifunctional accessory receptor with two mechanistically separable contributions to T cell immunity. CD2 stabilizes cell-cell contact through adhesion to CD58 (lymphocyte function-associated antigen 3; LFA-3) on opposing cells and transmits costimulatory signals from its intracellular tail following ligation. Unbiased tumor-cell genome-wide CRISPR screens performed in tumor and T cell coculture systems under selective pressure from either transgenic T cell receptor (TCR) T cells or CAR T cells identified CD58 as an essential tumor-intrinsic determinant of effective T cell-mediated immunotherapy, with CD58 loss impairing functional T cell responses.^5^^,^^6^ Additionally, pretreatment tumor CD58 expression is positively correlated with clinical outcomes in relapsed or refractory patients with large B cell lymphoma (LBCL) following anti-CD19 CAR T cell therapy.^7^ These data support defective CD2-CD58 engagement as a clinically meaningful resistance pathway.

CD2 function can be augmented through engineering approaches that either increase CD2 expression and pathway availability at the cell surface or bypass CD58 dependence by rewiring CD2-tail signaling. Colleagues and I recently described a PD-1:CD2 switch receptor that decouples CD2 signaling from CD58 availability by converting PD-L1 engagement into intracellular CD2 signaling.^8^ When co-expressed with a second-generation CAR, PD-1:CD2 improved *in vivo* activity in settings where the CD2:CD58 axis is compromised. Quantitative analyses of accessory receptor engagement by transgenic TCR T cells or CAR T cells show that CD2- and LFA-1-mediated signaling can provide the largest gains in antigen sensitivity and effector output, supporting a major contribution of CD2 signaling to functional performance.^9^ The downstream differentiation consequences of CD2 costimulation are directly relevant to therapeutic design, as CD2 agonism can attenuate exhaustion of chronically stimulated T cells and imprint a transcriptional program associated with favorable outcomes in infection and vaccination. This provides a mechanistic link between CD2 signaling and non-exhausted T cell states associated with therapeutic benefit.^10^

In our study, transient mRNA CAR expression enabled comparisons of receptor-proximal signaling while minimizing confounding effects from activation and expansion, whereas lentiviral CAR expression supported durability and *in vivo* testing.^4^ In the mRNA format, CD2ζ increased antigen-dependent proliferation relative to ζ-only CAR T cells and preserved degranulation and killing competence relative to signaling-deficient CAR T cells. CAR surface expression was broadly comparable across matched constructs, including CD28ζ and 4-1BBζ. Upon engagement with recombinant mesothelin, CD2ζ CAR T cells exhibited an intermediate Ca^2+^ flux phenotype, with faster decay than CD28ζ, whereas 4-1BBζ showed little to no flux above baseline. Across mesothelin and Tn-MUC1 platforms, CD2ζ maintained potent cytolytic activity while producing a more restrained acute cytokine output than CD28ζ, uncoupling tumor cell killing from maximal cytokine release. Transcriptional and phenotypic profiling further supported retention of less-differentiated T cell states and reduced enrichment of exhaustion-associated programs after stimulation.

**Figure 1:**
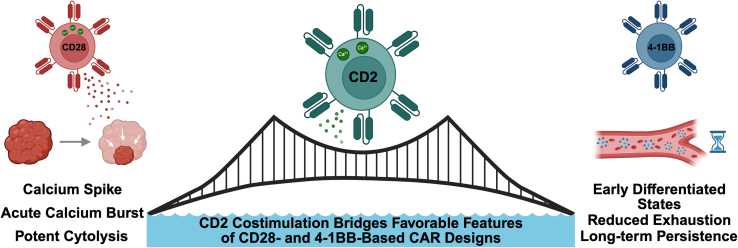
CD2 costimulation bridges favorable features of CD28-and 4-1BB-based CAR designs Second-generation CARs that incorporate CD28 (left) are commonly associated with high-amplitude early activation and rapid tumor debulking but can be accompanied by terminal effector differentiation and exhaustion under sustained stimulation. CARs that incorporate 4-1BB (right) more often support durability and persistence but may exhibit slower early cytolytic kinetics in settings that demand rapid tumor reduction. The CD2 cytoplasmic tail (center), when used as a CAR costimulatory endodomain, integrates advantages of both paradigms by tuning receptor-proximal signaling dynamics (intermediate Ca^2+^ signaling) while preserving cytolytic capabilities and promoting longer-lived functional states. Tumor cytolysis (left) and peripheral persistence over time (right) summarize the intended therapeutic outcomes of a CD2ζ-based CAR design strategy.

These features translated into durable antitumor activity *in vivo*. In mesothelioma and orthotopic pancreatic cancer xenograft models, CD2ζ CAR T cells drove deep tumor regressions with early clearance kinetics comparable to CD28ζ, while maintaining peripheral persistence more consistent with 4-1BBζ.^4^ Collectively, these data position CD2ζ as a signal-quality costimulatory domain that improves T cell fate control relative to canonical CD28- or 4-1BB-based costimulation, particularly under conditions that demand early debulking without forfeiting persistence (Figure 1).

Key next steps include mapping CD2ζ performance across antigen density and affinity landscapes to define activation thresholds and killing-persistence tradeoffs. Tumor CD58 expression should be evaluated as a biomarker-guided design variable to clarify when impaired CD2-CD58 engagement limits benefit and whether CD2ζ signaling can partially or completely compensate. Finally, CD2ζ CAR T cells should be tested in more stringent immunocompetent models that better capture stromal barriers and myeloid-driven suppression.

## Declaration of interests

A.D.P. is an inventor on patents and patent applications related to CD2-based CARs, mesothelin-specific CARs, and TnMUC1-specific CARs. A.D.P. receives royalties from Tmunity/Kite, a Gilead Company, for licensing of TnMUC1-specific CARs, and funding from Tmunity/Kite, a Gilead Company, for research on TnMUC1-specific CARs.
